# A Penance to Misdeed: A Case Report About Placenta Percreta

**DOI:** 10.7759/cureus.24399

**Published:** 2022-04-22

**Authors:** Monalisa Sarkar, Hemant Deshpande, Madhukar Shinde, Chetan Gulati

**Affiliations:** 1 Obstetrics and Gynecology, Dr. Dnyandeo Yashwantrao Patil Medical College And Hospital, Pune, IND; 2 Obstetrics and Gynecology, Dr Dnyandeo Yashwantrao Patil Medical College And Hospital, Pune, IND

**Keywords:** placenta, bishop score, manual removal of placenta, caesarean section, placenta percreta

## Abstract

Placenta percreta is a condition in which the placenta penetrates through the myometrium and into the uterine serosa. Sometimes it can be complicated by attachment to the surrounding structures or organs. The incidence of this condition is on the rise because of increased rates of cesarean sections. A 29-year-old gravida two, para one, living one with a previous cesarean section at 39 weeks of gestation was referred from a primary health center with complaints of leaking per vaginum for ten hours. She had no other associated symptoms. Her antenatal period was uneventful with all routine investigations within normal limits. A growth scan was done at 34 weeks of gestation. The scan was corresponding to the period of gestation with adequate liquor and placenta at fundoposterior location. A decision was taken to perform an emergency cesarean section in view of the previous cesarean section with premature rupture of membranes (PROM) and poor bishop score. After delivery of the baby when the placenta was tried to be removed, it could not be removed even with gentle traction. No plane of cleavage was identified between the uterine wall and placenta. The uterus was exteriorized and the placenta was found to have firmly adhered to the uterine wall and serosa on the fundal region. An intraoperative diagnosis of morbidly adherent placenta was made. A decision to perform an emergency obstetric hysterectomy was taken. A subtotal hysterectomy was done after counseling and necessary informed consent. Histopathology of the specimen was consistent with the findings of placenta percreta.

## Introduction

The placenta is a temporary endocrine organ found during pregnancy that secretes progesterone, estrogen, human placental lactogen, human chorionic gonadotropin, relaxin, etc. The placenta usually separates within 30 mins of delivery. If it does not separate then it may be morbidly adherent placenta. This implies abnormal implantation of the placenta into the uterine wall which includes placenta accreta, increta, and percreta. This is also known as the placenta accreta spectrum. Complete or partial absence of deciduas basalis leads to abnormal invasion of the chorionic villi into the myometrium. Placenta percreta is the rarest and most dangerous form because of its propensity to cause life-threatening hemorrhage.

The incidence of placenta percreta is on the rise due to increasing rates of cesarean deliveries [1]. The other risk factors are placenta previa, artificial reproductive techniques, previous history of placenta accreta, and previous uterine procedures such as curettages. The placenta accreta spectrum is usually seen in the lower uterine segment scars. After surgery, the scarring process leads to abnormal vascularisation and secondary hypoxia leading to defective decidualization and abnormal trophoblastic invasion.

Mostly it is asymptomatic. Antenatally it is diagnosed by the absence of uterine serosa-bladder interface on ultrasound and increased placental vascularity (placental lakes [2]) on color doppler. MRI is done if the ultrasound report is inconclusive or to rule out bladder bowel involvement. A multidisciplinary approach is needed for the optimization of maternal outcomes. A planned cesarean section with obstetric hysterectomy reduces maternal morbidity and mortality. Conservative management can also be tried but the rate of secondary hysterectomy is usually high due to a series of life-threatening complications like postpartum hemorrhage, sepsis, deep vein thrombosis, pulmonary thromboembolism, septic shock, fistulas, etc. We hereby present a case of an intraoperatively diagnosed case of placenta percreta and its successful management. In addition to the rarity of the condition, the location of the placenta percreta was also intriguing. This case has been presented at the Pune Obstetrics and Gynaecological Society (POGS) rotating trophy conference on 21 September 2021.

## Case presentation

A 29-year-old gravida two, para one, living one with the previous cesarean at 39 weeks of gestation was referred from a nearby primary health care center with complaints of leaking per vaginum for ten hours. She had no other associated symptoms. Her antenatal period was uneventful with all routine investigations within normal limits. An anomaly scan was done at 19 weeks and was found to be normal. A growth scan done at 34 weeks was found to be corresponding to the period of gestation with adequate liquor and placenta at fundoposterior location. She had a previous cesarean section done five years ago in view of fetal distress. No other significant past medical or surgical history.

On examination, she was vitally stable. Systemic examination was normal. On per abdominal examination, the uterus was found to be full-term, relaxed, clinically liquor appeared to be less, fetal heart rate was one forty beats per minute and regular. Per speculum examination demonstrated frank leak which was clear. On per vaginal examination os was one centimeter dilated, minimally effaced and the cervix was found to be posterior.

**Figure 1:**
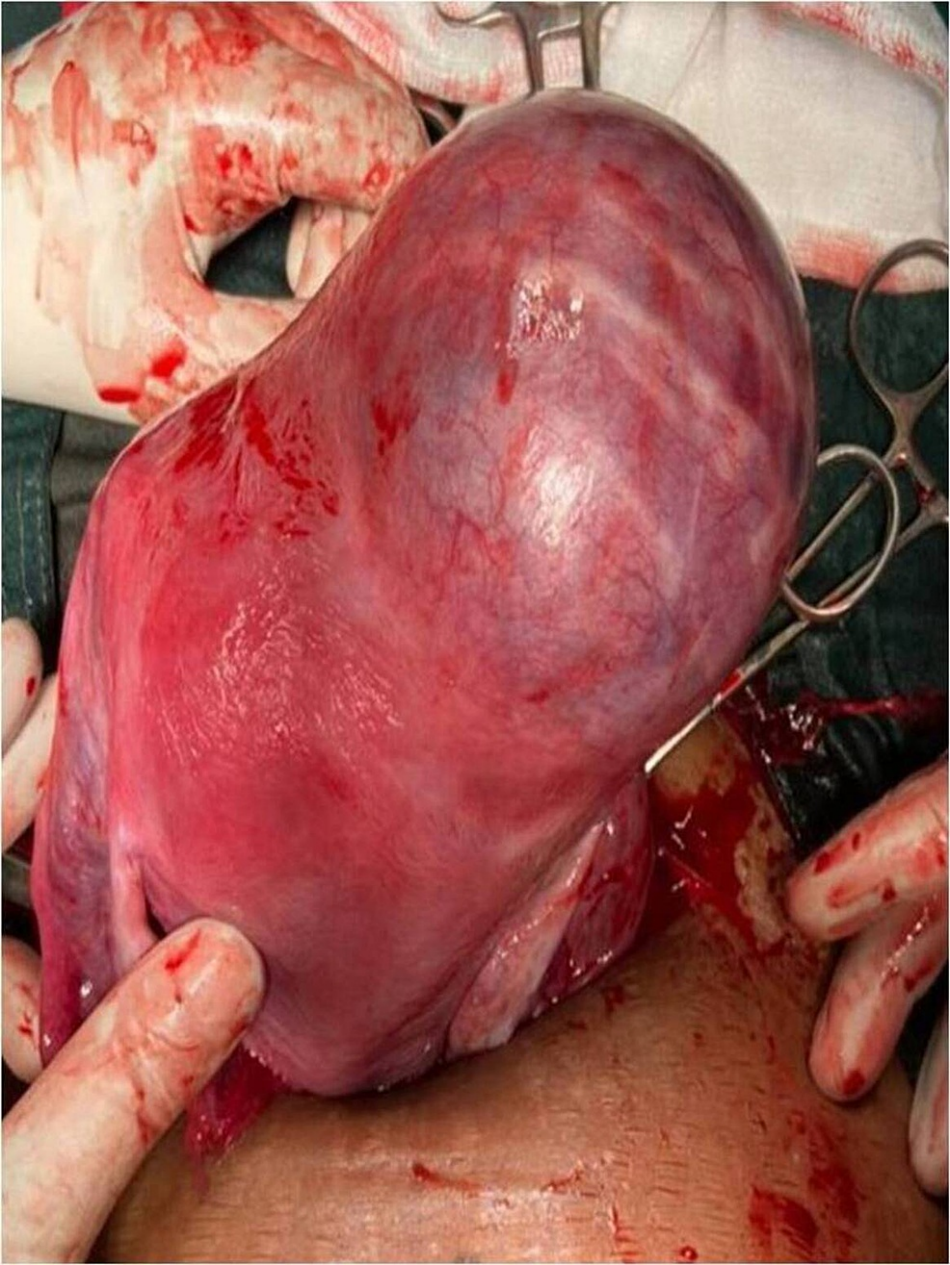
Placenta seen fundoposteriorly invading the serosa

A decision to perform an emergency cesarean section was taken. The indication was a previous cesarean section with prelabour rupture of membranes and poor bishop’s score. The abdomen was opened through a previous transverse scar under spinal anesthesia. The uterovescical pouch was opened and the bladder was pushed down. An incision was made in the lower uterine segment and a healthy male child of 2.6 kg was delivered. The placenta, however, could not be removed with gentle traction, and no plane of cleavage could be identified between the uterine wall and the placenta. The uterus was exteriorized and the placenta was found to have firmly adhered to the uterine wall and serosa on the fundal region (Figure 1). An intra-operative diagnosis of morbidly adherent placenta was made.

**Figure 2:**
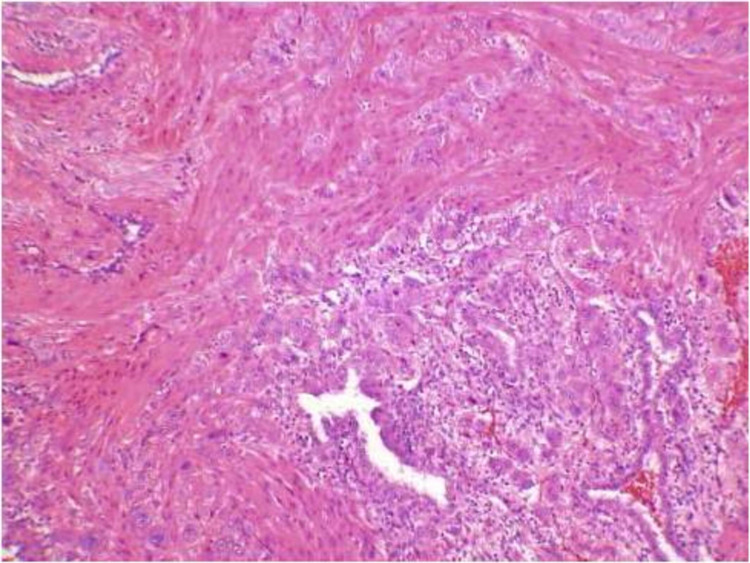
Microscopic examination of the specimen

A decision to perform an emergency obstetric hysterectomy was taken. A subtotal hysterectomy was performed after counseling and taking informed consent of the relatives. The patient’s vitals and urine output were monitored constantly. The post-op period was uneventful and the patient was discharged with a healthy baby after one week. Histopathology of the specimen was consistent with placenta percreta (Figure 2).

The morbidly adherent placenta usually has a propensity to occur at the previous cesarean scar site (i.e lower uterine segment) but in our case, it was seen fundoposteriorly. This is what makes our case unique. This draws our attention to the fact that manual removal of the placenta in her previous cesarean section could have been the reason for the unnatural location of the placenta percreta. Routine manual removal of the placenta can damage the basal layer of the decidua and lead to complications in future pregnancies as probably happened in our case. Hence the practice of manual removal of the placenta should be discouraged and let it deliver on its own. This is an important message which we want to convey to all practicing surgeons through our article. Our case also highlights the unexpected and unplanned intraoperative recognition of placenta percreta and its life-saving management.

## Discussion

When the placental villi are partially or completely attached to the underlying myometrium, it is termed placenta accrete. It occurs as a result of a defect in the deciduas basalis or defective development of nitabuch layer resulting in an abnormal invasion of the placenta. The term ‘accreta’ or ‘morbidly adherent placenta’ includes superficial invasion (accreta), myometrial invasion (increta), and serosal invasion (percreta). The incidence of placenta accrete has increased ten-fold in the past fifty years due to the increasing number of cesarean sections and maternal age. It now occurs with an approximate frequency of one in two thousand five hundred deliveries [3].

Though rare, it is now an important cause of maternal morbidity and mortality. It is one of the leading causes of peripartum hysterectomy. Patients with previous cesarean section/s and an antepartum diagnosis of placenta previa are at the highest risk of placenta accrete. The risk increases with the number of previous cesarean sections. Other risk factors include previous uterine surgery (like curettage, myomectomy), submucous fibroids, uterine anomalies, and asherman’s syndrome. Women with previous cesarean scar found to have a placenta previa or an anterior placenta underlying the scar must undergo additional diagnostic imaging to confirm or exclude placenta accreta. Though greyscale ultrasound is still a basic imaging modality for the diagnosis of morbidly adherent placenta, newer ultrasound techniques like color Doppler and three-dimensional power Doppler have improved the positive and negative predictive value and the diagnostic accuracy [4]. Loss of retroplacental sonolucent zone behind the placenta, placental lakes, or lacunae are suggestive of morbidly adherent placental MRI is a useful adjunct to ultrasound, especially in cases of the posterior placenta. MRI should be performed in all cases with an inconclusive ultrasound or Doppler and doubtful parametrial invasion.

Elevated levels of maternal serum creatinine kinase,alpha-fetoprotein, and beta-hCG have also been reported with placenta accreta [5,6]. The treatment for placenta percreta is primarily surgical with hysterectomy being the treatment of choice. With an expected placenta percreta, planned delivery at a tertiary care facility between 34 and 37 weeks is desirable with the availability of a multidisciplinary team [7]. Classical cesarean section (with no attempts of placental removal) with hysterectomy is the treatment of choice provided future fertility is not a concern. In case of future fertility is desired, after extensive counseling the conservative management may be tried.

Conservative management is desirable in rare cases which includes leaving the placenta in situ, uterine or internal iliac artery ligation and transcatheter arterial embolization have also been tried. Postoperative administration of methotrexate and arterial embolization has been tried in many patients to prevent bleeding and infection but is not always successful. Placenta insitu either undergoes spontaneous resorption or expulsion, which may take up to several weeks. Serious complications may occur which include DIC, uterine necrosis, fistula, peritonitis, sepsis, septic shock, acute renal failure, pulmonary edema, deep vein thrombophlebitis, or pulmonary embolism, and death. A multidisciplinary team approach is relevant in managing these patients to reduce morbidity and mortality.

## Conclusions

Placenta percreta is an extremely rare condition but an important cause of maternal morbidity and mortality. The incidence is on the rise due to the increasing number of cesarean deliveries. Hence doctors should be more careful and have a genuine rationale for the performance of cesarean sections. As antenatal suspicion and diagnosis can be life-saving for a patient, therefore clinicians and radiologists should be more vigilant. Our case also teaches us to avoid the malpractice of manual removal of the placenta to avoid complications in future pregnancies.
